# Decarbonising road freight transport: The role of zero-emission trucks and intangible costs

**DOI:** 10.1038/s41598-024-52682-4

**Published:** 2024-01-24

**Authors:** Vahid Aryanpur, Fionn Rogan

**Affiliations:** 1 SFI MaREI Centre for Energy, Climate and Marine, Environmental Research Institute, University College Cork, Cork, Ireland; 2 School of Engineering and Architecture, University College Cork, Cork, Ireland

**Keywords:** Energy science and technology, Environmental sciences

## Abstract

The road freight sector faces significant challenges in decarbonisation, driven by high energy demand and limited availability of low-emission fuels and commercialised zero-emission vehicles. This study investigates intangible costs associated with advanced electric and hydrogen-powered trucks, including recharging/refuelling time, cargo capacity limitations, and buyer reluctance towards emerging technologies. Utilising a comprehensive whole-systems modelling approach considering low- and zero-emission fuels, inter-sectoral dynamics, and the carbon budget, we explore cost-optimal decarbonisation pathways for heavy, medium, and light trucks. Scenario and sensitivity analyses reveal the following insights: (1) Electric trucks dominate the market under mitigation pathways across all weight categories. However, the inclusion of intangible costs triggers a shift, leading to the emergence of hydrogen fuel cell vehicles for heavy trucks, while battery electric vehicles are preferred for medium and small trucks. (2) Prioritising heavy truck decarbonisation and taking early action are crucial to avoid carbon lock-in effects. (3) Considering limited decarbonisation options, where electric and hydrogen-fuelled trucks are pivotal, this research highlights the significance of policy instruments targeting operational expenditures over conventional purchase price incentives. Such policies offer dual benefits by supporting truck owners and directing incentives more precisely towards achieving measurable emission reductions.

## Introduction

Between 2000 and 2022, there has been a notable 55% increase in global tailpipe CO_2_ emissions from the road freight sector, contributing 5% to the total worldwide energy-related CO_2_ emissions during this period^1^. Despite accounting for only 9% of the global vehicle stock and 17% of total vehicle miles driven, freight trucks are responsible for approximately 39% of life-cycle road vehicle Greenhouse Gas (GHG) emissions^2^, with even higher shares for other air pollutants^3^. The strong link between road freight and economic growth^4^ has led to projections indicating a near doubling of freight transport activities by mid-century^5^. Given their disproportionate emissions contribution and expected growth, climate scientists, energy and transport planners are closely examining the implications^6^ across different countries.

Ireland, among other countries, has pledged to reach a legally binding target of net zero GHG emissions no later than 2050^7^. The transport sector poses a significant challenge as emissions in this sector have risen by 115% during the past two decades^8^. To achieve a carbon neutral target, it is crucial to decarbonise the transport sector and particularly the hard-to-abate Heavy Goods Vehicles (HGVs). Similar to global statistics, HGVs make disproportionate contribution as they make up 5% of total road vehicles^9^, but they generate more than 21% of the total CO_2_ emissions from the transportation sector^8^. The overall freight demand is projected to double by mid-century^10^ in Ireland, making the task of reducing emissions in this sector increasingly challenging^11^.

It is widely acknowledged that the transition of heavy goods freight is complex^12^ because many of the alternatives currently have a low level of technological readiness and infrastructure availability^13–16^. Battery electric vehicles (BEVs) and hydrogen Fuel Cell Vehicles (FCVs) are the most promising technologies to fully avoid tail-pipe emissions from HGVs^17^. These vehicles are categorised as Zero Emission Vehicles (ZEVs). Direct electrification using BEVs has substantial efficiency advantages over hydrogen use due to the energy conversion losses in production and utilisation of hydrogen in FCVs^18^. Despite the development of numerous vehicle models, particularly for medium freight trucks^19^, electrification is not currently a viable option for larger vehicles that are often used for long-distance transportation^20^. Fuel cell technology for heavy-duty vehicles is still in the early stages of development and deployment^20^. Despite this, FCVs have superior range capability in comparison to BEVs^21^. Additionally, their refuelling times and cargo capacities^22^ are comparable to conventional Internal Combustion Engines (ICEs). Moreover, electricity^23^ and hydrogen supply^24^ infrastructures are critical to support the adoption of BEVs and FCVs. A thorough survey of European fleet operators, logistics providers and shippers, and business associations reveals that the immaturity of technologies and their associated infrastructures are the primary roadblocks to a faster transition, preventing the widespread adoption of zero-emission trucks^25^. This results in freight and haulage representative organisations and companies being hesitant to make decisions, leading to delays in the transition to more sustainable transportation options and putting carbon reduction goals at risk^26^.

Several previous studies have thoroughly examined sustainable and eco-friendly approaches for road freight transportation. They focused on ZEVs specifically designed for heavy-duty transport, while also exploring the integration of zero-emission fuels (ZEF) as viable options for trucks. Lajevardi et al*.*^27^ compared the GHG emissions and abatement costs of ZEVs with incumbent drivetrains and suggested FCVs for short haul routes. Gunawan and Monaghan^28^ found that battery electric trucks powered by a highly renewable electricity grid are both environmentally friendly and have low ownership costs. Ruhnau et al*.*^29^ investigated the potential benefits of direct and indirect electrification, specifically the use of grid electricity to power BEVs and the production of hydrogen and methane from electricity to drive FCVs. They concluded that as efforts to reduce emissions increase, the shift towards electrification of road transport is expected to become greater. Yet, the scenarios reviewed show no clear preference for either direct or indirect electrification of truck transport. Simulation by Giuliano et al*.*^30^ suggests that the short-term viability of BEVs is hampered by charging and range limitations. However, as battery performance improves and prices decrease, their study highlights the potential for a radical market uptake of BEVs. Çabukoglu et al*.*^20,31^ concluded that FCVs have a higher technical potential than BEVs because of their longer range. However, their findings should be viewed with caution as they assumed that the required hydrogen infrastructure was already in place. Despite valuable insights from this group of studies, they have solely examined the decarbonisation of HGVs through single-sector analysis, neglecting cross-sectoral interactions.

Energy Systems Optimisation Models (ESOMs) can be used to understand the complex interactions and capture dynamics across the entire energy system. ESOMs are widely used to inform national-level decision-making^32^. These technology-rich, bottom-up models use linear programming to minimise the cost of energy provision by optimising technology capacity and utilisation^33^. Using ESOMs, previous studies have explored a range of topics, including the introduction of FCVs^34^, travel behaviour and travel time budget^35^, improving behavioural realism of vehicle users^36^, and mitigation potential through higher biodiesel blend ratio^37^. However, these studies have mainly focused on passenger transport sector and often treated the HGVs, in an aggregated manner. The aggregation may limit detailed analysis of the challenges and opportunities of decarbonising freight sector. A few energy systems modelling studies disaggregated the transportation segment to specifically consider trucks as a separate category^38,39^, or various modes of transportation are broken down further according to driving patterns^40^ and freight capacity^41^. Despite further details, they have not captured the intangible costs associated with the adoption of ZEVs.

Intangible costs represent non-financial factors influencing vehicle purchasing decisions, including availability, reliability, quality, social desirability, and popularity among operators and drivers^16,42^. Hao et al.^43^ developed a perceived cost of ownership model that revealed implicit costs related to range anxiety and charging inconvenience constitute at least 27% of the perceived cost of ZEVs, making them uncompetitive. Hammond et al.^44^ estimated intangible costs increase the capital cost of ZEVs by about 40%.

In summary, the challenge in reducing emissions from HGVs is mainly because of the high energy demand of trucks and the limited low-carbon alternatives available in the short term. The long lifespan of HGVs also complicates rapid emissions reductions. Without a comprehensive understanding of the technology landscape and its consequences, incorporating sustainability into long-term plans may become challenging. Furthermore, if the reduction of emissions from the road freight sector is not effectively tackled, it can compromise national carbon budgets. Postponing action to achieve decarbonisation pathway will result in a quicker utilisation of the carbon budget by easier-to-abate sectors. This will leave hard-to-abate sectors, including road freight, with less time to implement necessary infrastructure and investments for decarbonisation^45^. This can also result in failure to meet emissions targets or make carbon neutrality more difficult and expensive in the future. The current study fills the gap in exploring decarbonisation pathways for HGVs by using a whole-systems modelling approach that accounts for inter-sectoral dynamics, intangible costs, and the carbon budget.

This research makes a novel contribution to the road freight sector’s net zero emission goals by endogenising the intangible costs of adopting BEVs and FCVs. It incorporates the hidden costs of using zero emission trucks, including significant recharging/refuelling time, reduced cargo capacity, and reluctance to invest in immature technology by vehicle buyers. The study distinguishes itself by utilising a system-wide modelling approach that draws attention to previously disregarded obstacles, including hydrogen and electricity supply systems. Additionally, it disaggregates HGVs into three categories: light, medium, and heavy, allowing for customised solutions tailored to the unique characteristics of each category. It provides a consistent accounting framework for specifying the techno-economic performance of various technologies including sectoral interactions. The model also ensures net zero emissions across the entire energy system using carbon budget approach. The key objectives of this study are to: **(1)** identify the feasible pathways to decarbonise the road freight sector, **(2)** assess the impact of intangible costs on the adoption of ZEVs in various weight categories, **(3)** analyse the effect of zero-emission trucks on the energy system, with a focus on electricity and hydrogen supply, and associated costs. While this paper explores the decarbonisation potential of various vehicle technologies for HGVs in Ireland, we derive generic insights that are applicable and valuable for national policy-making across different countries. By addressing common concerns and uncertainties related to technology adoption and market incentives, this research contributes to a global dialogue that seeks to foster sustainable practices in the road freight sector.

## Results

This section provides optimal fleet and fuel mix and the corresponding CO_2_ emissions in different scenarios. Then the results of sensitivity analysis are explored.

### Fleet mix

**Figure 1:**
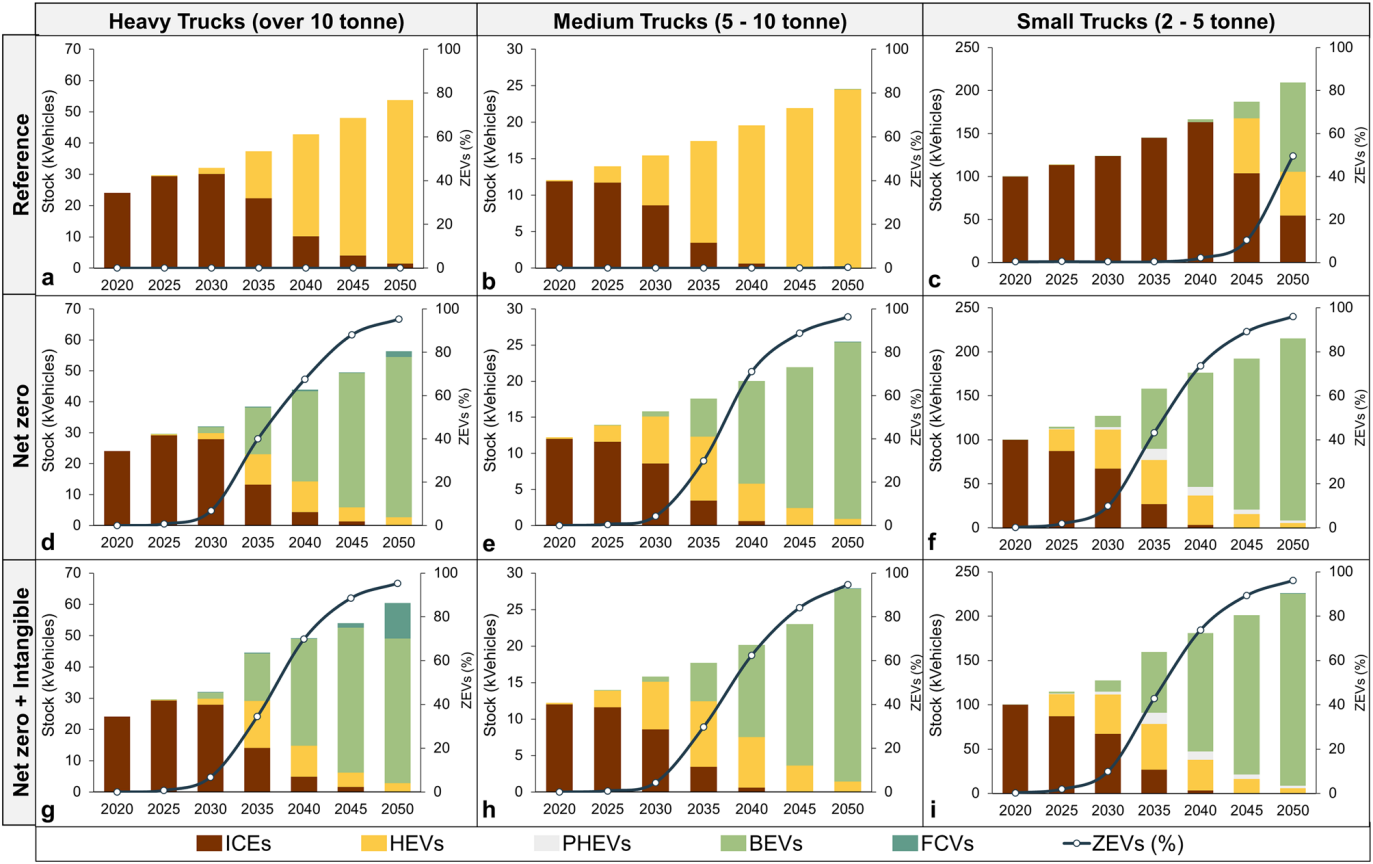
Fleet mix in different scenarios and for different weight categories.

Figure 1 shows the changes in fleet mixes for light, medium, and heavy trucks across various scenarios over the next three decades.

As depicted in Fig. 1a,b, in the reference scenario, the ICEs are gradually replaced by Hybrid Electric Vehicles (HEVs) within the heavy and medium truck sectors. This market shift aligns with the model’s least cost logic, which emphasises the preference for more efficient vehicles that maintain a similar technological framework. Moreover, it avoids incurring significantly higher upfront costs associated with advanced vehicles, as well as the infrastructure for hydrogen and electricity supply. The adoption of ZEVs remains minimal over the planning horizon. For small trucks in Fig. 1c, the cost-optimal results reveal the gradual adoption of BEVs starting from 2040. By 2050, BEVs dominate the small truck fleet. This trend can be attributed to the anticipated advancements in battery technology, which are expected to be more feasible for small trucks.

By implementing carbon budget constraints in the Net Zero (NZ) scenario, there is a significant transformation in the market dynamics, particularly towards electrified freight transportation across all weight categories. As illustrated in Fig. 1d,e,f, BEVs emerge as the predominant choice, exceeding 95% of the total market share across different truck weights. HEVs are also identified as a viable mid-term solution for all weight categories. Additionally, Plug-in Hybrid Electric Vehicles (PHEVs) are recognised to have a substantial role in facilitating the transition towards a decarbonised pathway for light trucks. FCVs emerge in the last period with a marginal market share for heavy trucks.

By activating intangible costs in the Net Zero plus Intangibles (NZI) scenario in Fig. 1g,h,i, the transition follows a similar trend to the NZ scenario until 2040–2045. However, a divergence occurs for heavy trucks, as they undergo electrification first, followed by a substantial adoption of FCVs. This shift is driven by higher intangible costs associated with limited cargo capacity and reduced availability factor due to longer recharging times for BEVs. The model reflects the reduction in hydrogen supply costs and a decreasing trend in upfront costs of FCVs, leading to their increased market presence to around one-fifth of the market share. It is important to note that smaller trucks exhibit a similar fleet mix as observed in the NZ scenario. This can be attributed to the fact that intangible costs have limited impact on the fleet composition since battery technologies are expected to be fully commercialised for smaller trucks. This notion is supported by our assumptions that the limitations of small BEVs are expected to diminish at a faster rate compared to heavy trucks.

In the Reference scenario, the total number of heavy and medium trucks shows a gradual increase until 2030. However, from 2035 onwards, there is a substantial growth in truck numbers due to higher retirement rates and the replacement of low-efficiency ICE vehicles with more efficient trucks. In the NZ and NZI scenarios, the number of heavy trucks closely follows the Reference scenario until 2030. However, from 2035 onwards, the adoption of zero-emission trucks accelerates. Due to limited cargo capacity and prolonged recharging time, the total number of trucks in these scenarios increases at a faster pace to meet the demand.

### Fuel mix and CO_2_ emissions

**Figure 2:**
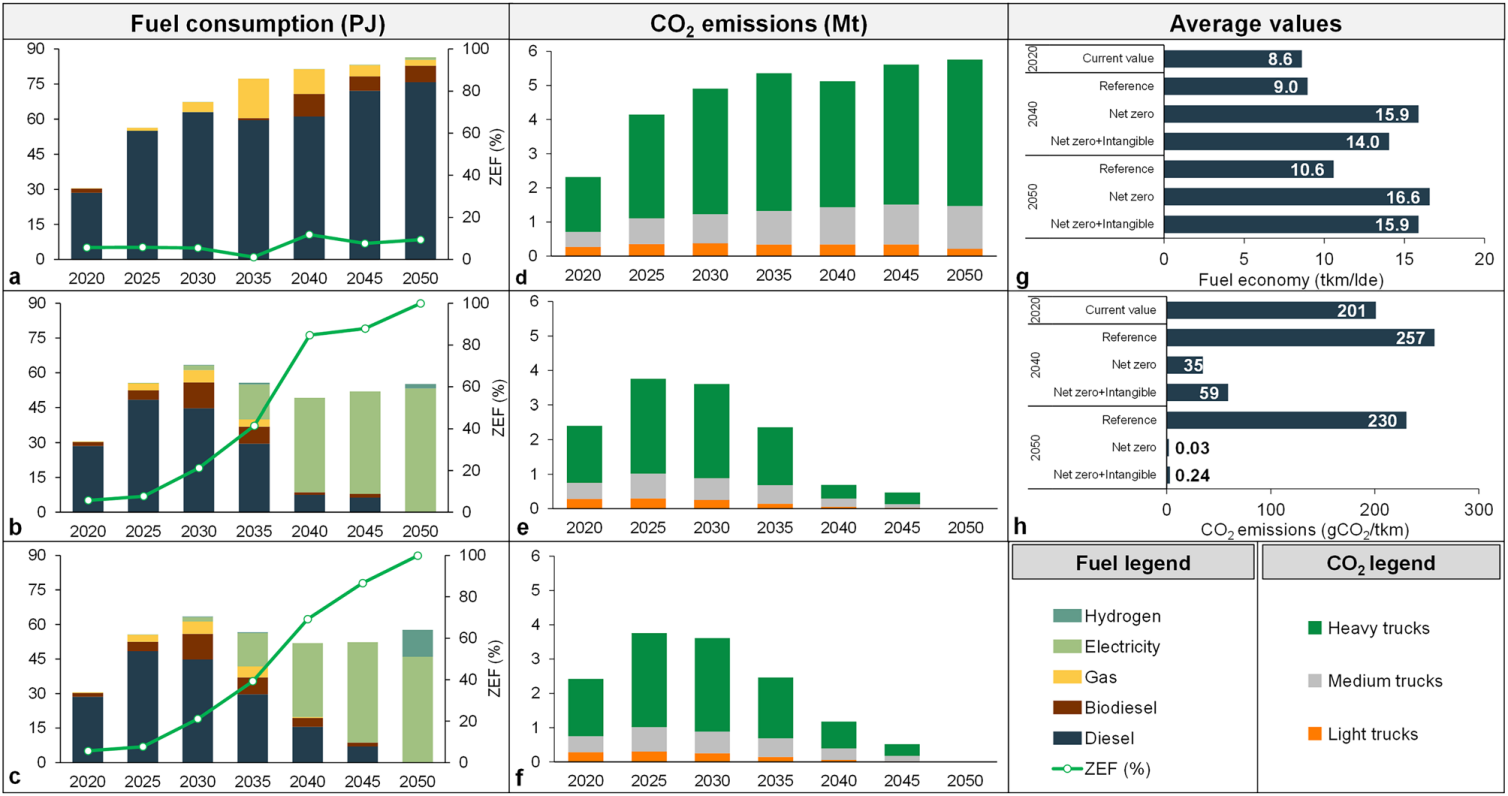
Fuel consumption and CO_2_ emissions in freight sector (Fuel consumption in (**a**) Reference scenario, (**b**) NZ scenario, (**c**) NZI scenario; CO_2_ emissions in (**d**) Reference scenario, (**e**) NZ scenario, (**f**) NZI scenario; (**g**) Average fuel economy (**h**) Average CO_2_ emissions).

Figure 2 illustrates fuel consumption for freight transportation and related CO_2_ emissions. In the Reference scenario in Fig. 2a, diesel fuel predominantly powers conventional ICE and HEVs. Biodiesel and natural gas also play a moderate role in the mid- to long-term. However, the share of ZEF, including electricity, hydrogen, and biodiesel, is projected to remain below 12% throughout the study period. In the mitigation scenarios (Fig. 2b,c), electricity and hydrogen play the key role, dominating the market by 2050. In 2020, COVID-19 restrictions significantly impacted transportation, leading to relatively low fuel consumption. The Reference scenario indicates radical fuel consumption over time due to reliance on less fuel-efficient ICE-based fleet mix. However, the NZ scenario, with increased adoption of BEVs, maintains stable fuel consumption and achieves a remarkable 36% reduction by 2050 compared to the Reference case. The NZI scenario, incorporating hydrogen and electricity as fuel sources, experiences a slight increase in fuel consumption compared to the NZ scenario but still accomplishes a significant 33% reduction compared to the Reference one. Figure 2g provides an additional perspective on the differences in average fuel economy, which is measured in tonne.kilometre per litre of diesel equivalent (tkm/lde). Compared to the base year, the average fuel economy improves by 23%, 93%, and 85% in the Reference, NZ, and NZI scenarios, respectively. This demonstrates how the transition to ZEVs and the adoption of more efficient technologies contribute to enhancing the fuel economy of the fleet.

Figure 2d,e,f provide a comparative analysis of CO_2_ emissions disaggregated by weight category across various scenarios. In the Reference scenario, CO_2_ emissions continue to escalate until 2035, after which they stabilise at a nearly constant level. It occurs as the increased demand for total tkm is offset by the adoption of more efficient electric and hybrid vehicles. In this scenario, the total emissions are expected to be more than double the initial value. However, it is not surprising that the alternative scenarios with carbon budget constraints demonstrate decarbonisation for whole weight categories. Figure 2h provides average CO_2_ emissions in grams per tonne.kilometre (gCO_2_/tkm). It shows the potential for significant emission reductions by transitioning to ZEVs and implementing decarbonisation strategies. One crucial aspect to highlight is that the model ensures decarbonisation across the supply-side of energy. This means that the electricity used in the scenarios is generated from a power system based on renewable sources, while the production of hydrogen utilises electrolysers powered by renewable-based electricity. This comprehensive approach ensures that the emissions associated with energy generation are minimised.

### Sensitivity analysis

In this section, the individual impacts of intangible costs are assessed. The assessment involves exploring various sensitivity cases where intangible costs deviate by ± 30% from the base year values in the NZI scenario. For simplicity, the results presented here focus on the heavy weight category, which is considered the most significant contributor to emissions in the freight sector.

**Figure 3:**
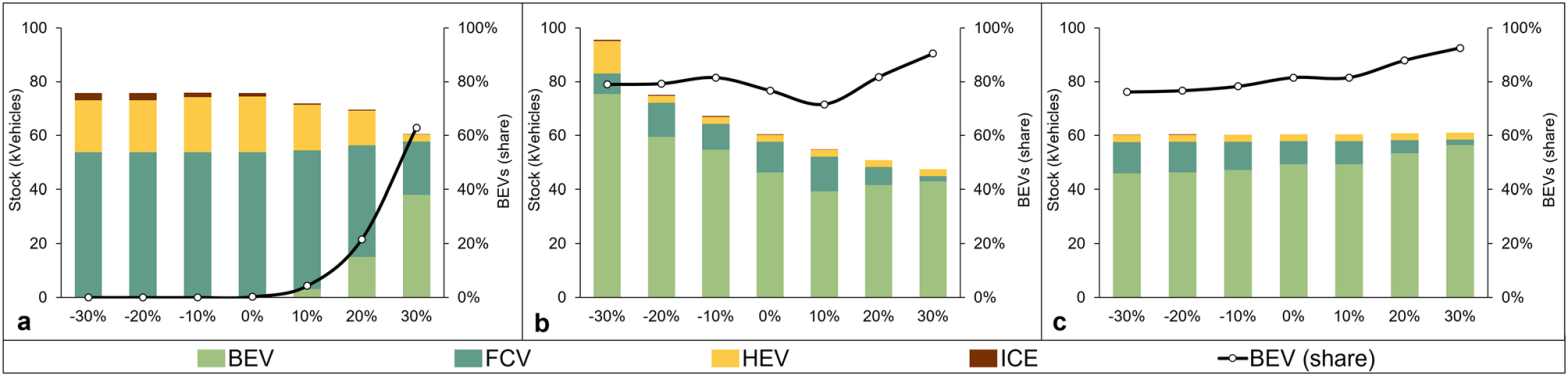
Sensitivity of vehicle fleet mix in 2050 by varying (**a**) BEVs’ cargo capacity, (**b**) BEVs’ recharging time, and (**c**) hurdle rates.

Figure 3a illustrates the impact of varying cargo capacity on BEV adoption in the total vehicle mix by 2050. In scenarios with − 30% to − 10% cargo capacity reductions, FCVs dominate the fleet, and BEVs are absent. However, as cargo capacity improves by 10%, BEVs gradually emerge and represent 4% of the total vehicles. Increasing cargo capacity by 20% and 30% results in a significant shift, with BEVs capturing 22% and eventually 63% of the total vehicles. This highlights cargo capacity as a key factor in driving zero-emission truck adoption. Additionally, increasing cargo capacity leads to a decrease in the total number of vehicles, as higher load factors enable fulfilling demand with fewer trucks, emphasising the benefits of higher cargo capacity.

Figure 3b illustrates the impact of varying BEV recharging time on the vehicle fleet mix in 2050. Prolonged recharging times result in diminished vehicle availability, particularly evident in the − 30% and − 20% cases, leading to a substantial surge in the total number of BEVs within the fleet. The constrained availability of BEVs necessitates a higher overall number of vehicles to meet demand. Despite the challenges posed by lower availability, BEVs continue to maintain a substantial share in the fleet, accounting for approximately 80%. This persistence is attributed to the cost-effectiveness of BEVs when compared to fuel cell technology, highlighting the significance of upfront investment requirements, particularly in developing hydrogen supply infrastructures, in shaping the adoption dynamics. Lifetime consideration is another factor, as BEVs adopted from 2030 to 2045 continue operating in 2050. However, the limited cargo capacity of BEVs, along with the need for further improvements in battery energy density^46^, remain important factors hindering their complete dominance in the market. Ongoing advancements, such as the development of next-generation batteries^47^, are crucial in driving wider adoption of BEVs.

Figure 3c demonstrates that the higher the hurdle rates, the greater the diffusion of BEVs in the vehicle fleet mix by 2050. However, the total number of vehicles remains insensitive to changes in hurdle rates, and overall fleet composition also shows limited variations across all cases. This aligns with the findings emphasised by Konstantinou and Gkritza^48^, highlighting the importance of government support and incentives to enhance the acceptance of electric vehicles among vehicle buyers.

## Discussion

This study addresses the decarbonisation pathways for HGVs. It employs a whole-systems modelling approach that considers inter-sectoral dynamics, intangible costs, and the carbon budget. While showcasing the results for Ireland, this study adds to the global discourse on promoting sustainable practices in the road freight sector by addressing common concerns and uncertainties related to technology adoption and market incentives. The key findings from this research are as follows:

Our results provide a crucial insight into the adoption of zero-emission trucks, highlighting that operational parameters have a more significant influence than the purchase price. Specifically, variations in cargo capacity and recharging time (both operational factors) strongly influence the size and composition of the vehicle fleet under mitigation scenarios, while hurdle rates (which primarily impact upfront costs) have a lesser impact. These findings are in line with the results of a comprehensive Total Cost of Ownership (TCO) analysis conducted by Noll et al*.*^49^ across 10 European countries. This European study emphasised the substantial influence of Operational Expenditure (OPEX) parameters on freight vehicle TCO outcomes. It showed that OPEX parameters contribute to approximately 75% of the TCO for all vehicle types in all countries, with the remaining portion attributed to Capital Expenditure (CAPEX). Contrary to the conventional approach, which primarily focuses on providing CAPEX subsidies to promote the adoption of alternative-drive vehicles, particularly for private cars^50^, the present research and the TCO comparison across EU countries suggest that OPEX incentives can be more effective for freight vehicles. This finding is also consistent with another techno-economic analysis conducted in the US market, emphasising that electric trucks demonstrate cost-effectiveness with increased utilisation compared to traditional counterparts^51^. As a result, policy instruments that target OPEX parameters are more likely to enhance the competitiveness of zero-emission trucks. It is worth noting that the limited options of BEVs and FCVs in the context of heavy truck decarbonisation highlight the need to carefully assess operational costs. While upfront cost reductions are expected for both technologies, their economic competitiveness will significantly be determined by operational factors such as fuel consumption and vehicle availability. While upfront cost incentives may seem straightforward and can be tailored to target specific groups (such as early adopters)^52^, OPEX incentive policies have the dual potential of benefiting truck owners and ensuring that incentives are precisely directed toward realising tangible emission reductions and making measurable contributions to environmental improvements. In fact, such policies strengthen the tie between financial supports and the actual performance of mitigation measures, thereby extending benefits beyond the boundaries of end-users.

Scenario analyses reveal two crucial facts for achieving successful mitigation. Firstly, heavy trucks are the primary contributors to emissions across all scenarios, underscoring the urgency of prioritising decarbonisation efforts for this weight category to achieve efficient carbon mitigation in the road freight sector. Secondly, emissions pathways in the mitigation scenarios emphasise the significance of early action to prevent lock-in effects and achieve cumulative emissions reductions, as mandated by carbon budgets.

The influence of intangible costs on the market adoption of heavy truck technologies suggests a preference for FCVs, while electric trucks are preferred once intangible costs are addressed. For smaller truck categories, even when intangible costs are taken into account, electrification remains the preferred option.

During the study period, despite the doubling of freight demand, the successful adoption of ZEVs results in a reduction of total fuel consumption by at least one-third. It highlights the significant potential of zero-emission trucks in curbing fuel consumption and enhancing overall fuel economy. They serve as a clear indicator of the need to transition towards zero-emission fuels and promote efficient technologies for a sustainable and environmentally-friendly freight sector.

Some caveats of this analysis should be acknowledged for future studies. First, daily travel behaviour’s impact is not considered in this study. However, for small and medium-sized trucks with limited daily mileage, drivers may manage recharging requirements effectively, favouring the adoption of BEVs over FCVs. Future research could also assess the potential impacts of contact-line electric road systems (overhead catenary), a mature solution with successful projects in Sweden and Germany^53^, and proven viability in British Columbia^27^. Another area for future studies could explore battery swapping as a solution to address prolonged recharging time^54^, considering logistical and economic complications and potential trade-offs with recharging infrastructure for all electric vehicles^55^. Second, cost optimisation modelling is highly sensitive to input parameters, particularly the upfront investment costs of emerging technologies. Limited changes can trigger a “knife-edge solution” or “penny switching effect” leading to significant shifts in technology preferences^56^. Future research should thoroughly address uncertainties related to upfront costs, aiming to uncover unexpected solutions and ensure a comprehensive understanding of the model’s outcomes. Third, the availability of vehicle models presents a significant barrier to electric trucks^48^. It is crucial to consider the technology readiness and manufacturing readiness of zero-emission trucks for series production, as these factors significantly impact large-scale ZEV adoption. Further research is needed to investigate market dynamics and provide accurate insights for energy modelling studies in this domain.

## Methodology and data

This section covers the structure of TIMES-Ireland Model (TIM), as well as the structure of HGVs within the model. Then different scenarios are defined.

### TIMES-Ireland model

TIMES (The Integrated MARKAL EFOM System) as a bottom-up, techno-economic optimisation model is used in this study. TIMES model is a powerful tool for analysing energy systems and evaluating pathways for decarbonisation and can be used to assess the cost and feasibility of different energy supply and demand options. By considering a range of technology options and their interconnections, the model provides insights into the optimal mix of technologies to achieve a low-carbon energy system. The optimal solution is the minimisation of the total costs of the entire energy system discounted to a base year^57^. The developers of TIMES have provided a detailed explanation of the source code, input data, and mathematical formulation in reference^58^.

**Figure 4:**
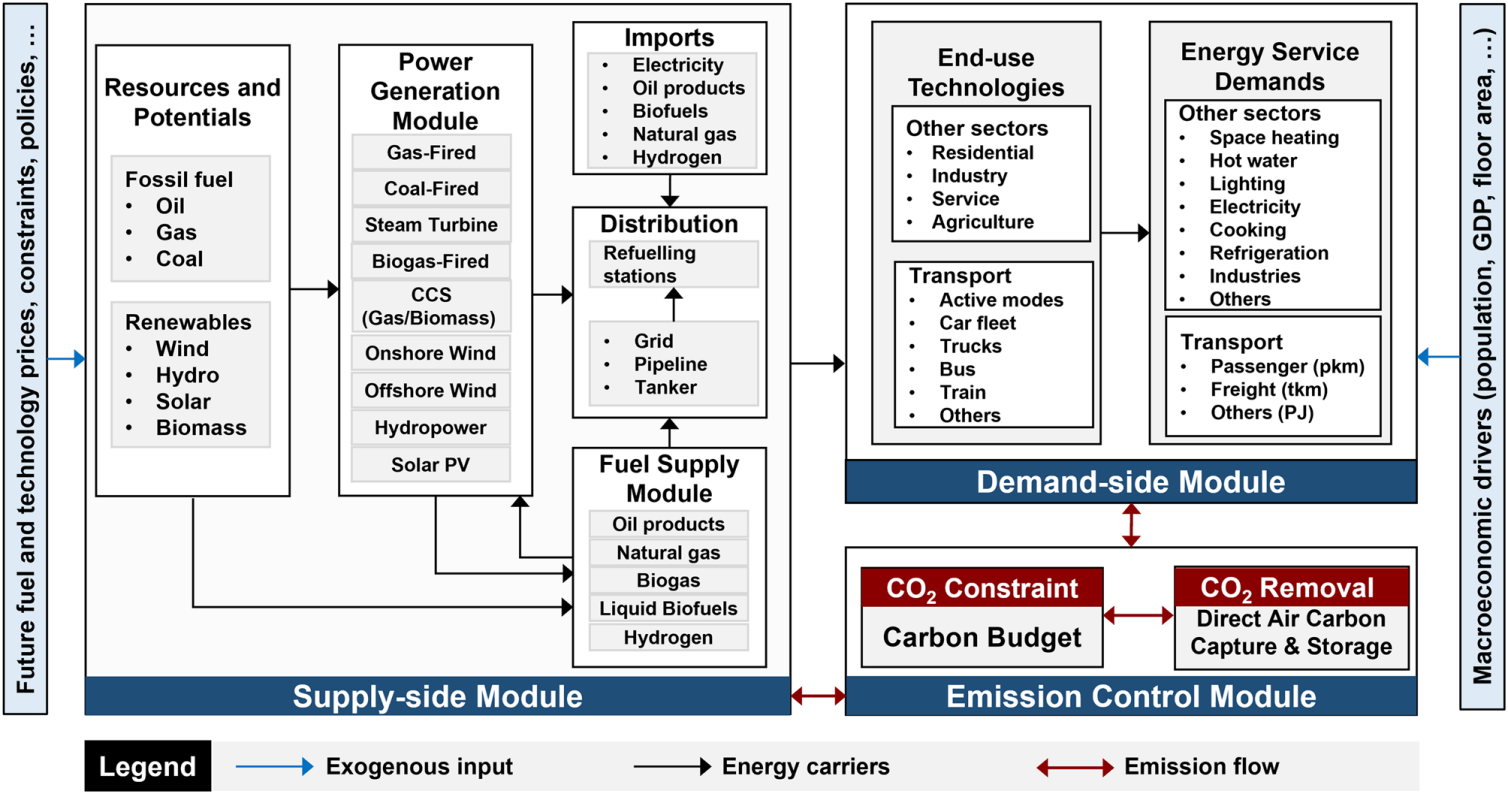
Simplified structure of energy system in TIM.

Figure 4 provides a simplified overview of the TIM, comprising three major components. The supply-side module covers various energy resources, fuel production, conversion technologies, and transmission infrastructure. Hydrogen production via centralised and decentralised electrolysis options are modelled. Delivery methods, including high-pressure transmission and distribution pipelines, as well as road tanker options, are analysed. Additionally, the TIM models hydrogen storage and dispensing for refuelling FCVs.

The demand-side focuses on energy service demands in different end-use sectors. The emission control module tracks CO_2_ emissions, ensuring compliance with carbon constraints and enabling carbon-neutrality through direct CO_2_ removal technology utilisation. The TIM is a well-established model comprehensively described in reference^59^ applied in light-duty vehicles decarbonisation^50^ low energy demand analyses^60^ and the decarbonisation pathways in the residential sector^61^.

### Road freight transport structure

**Figure 5:**
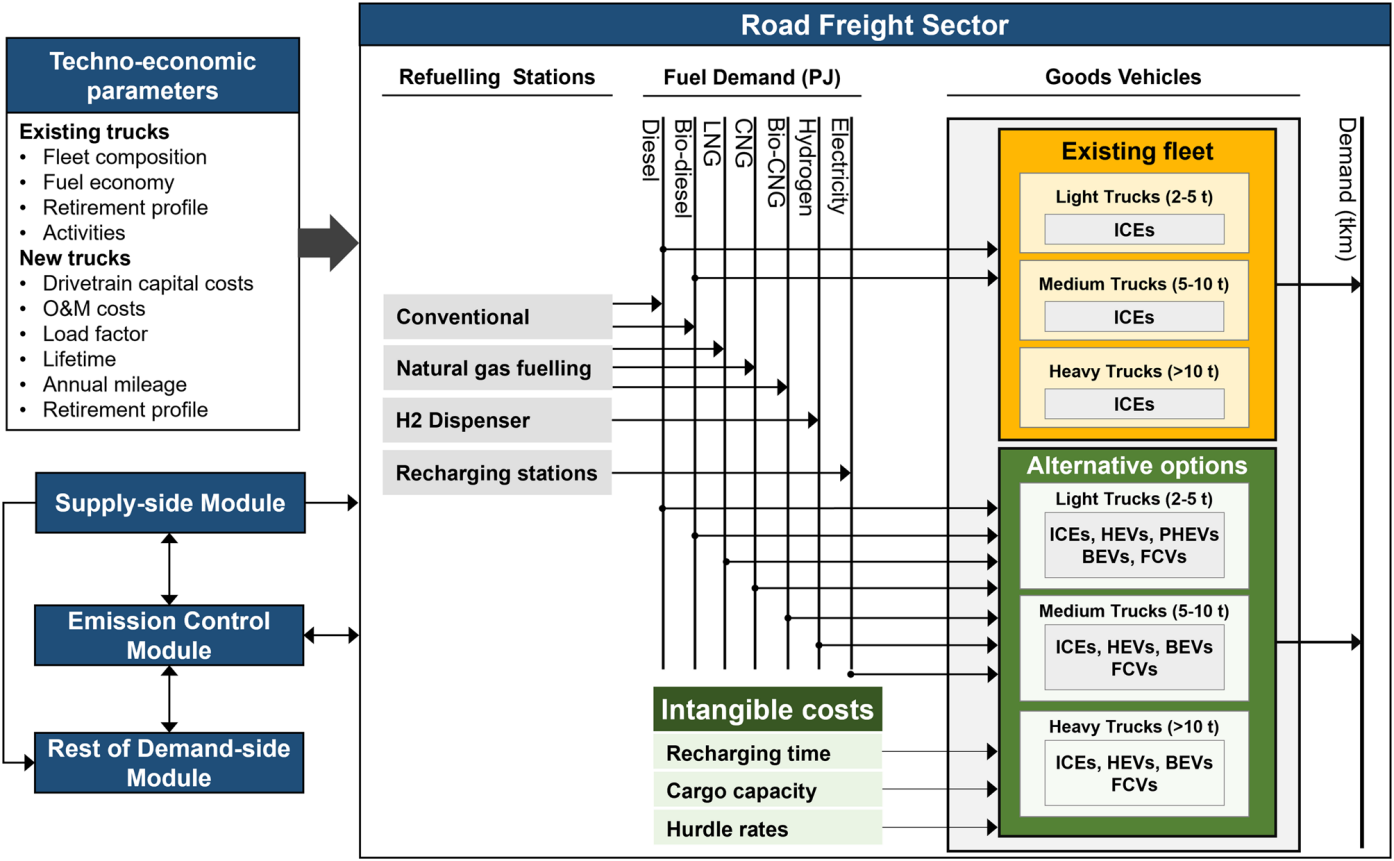
Structure of the road freight sector and its interactions with other sectors.

Figure 5 shows the detailed structure of the road freight transport sector analysed in this study and how it interacts with other components of the model. HGVs are classified into three categories based on their unladen weight: light trucks (2–5 tonnes), medium trucks (5–10 tonnes), and heavy trucks (over 10 tonnes). Currently, the existing fleet is powered by ICEs that run on diesel and biodiesel. An average national retirement profile is used to simulate the scrappage of the existing vehicles. For the future, we considered five groups of technologies: (1) advanced ICEs with higher fuel efficiency, using different types of fossil fuels, biofuels, and gas-based fuels; (2) HEVs with an ICE and a small electric motor; (3) PHEVs that have a similar powertrain to HEVs but can charge their batteries from the grid; (4) BEVs that rely solely on batteries charged from the electricity grid; and (5) FCVs that use a pressurised hydrogen storage tank and an electrochemical device to generate power for the vehicle’s electric motor. Distribution of fuels is through four types of typical refuelling stations: conventional liquid pumps, natural gas fuelling stations, hydrogen dispensers, and electric recharging stations.

### Intangible costs

Intangible costs for heavy goods vehicles refer to those indirect expenses that are not immediately and explicitly evident but have a significant impact on the operation of the freight transport sector. In this paper, three main intangible costs are addressed.

First, the reduced cargo capacity of zero-emission trucks like BEVs and FCVs is a significant challenge. The need for large battery systems and hydrogen tanks occupies additional space and adds weight, leading to lower freight volumes transported per vehicle. This can decrease the efficiency of freight transportation, requiring more trips to move the same cargo volume, resulting in increased fuel and labour costs^62^. Large batteries in electric vehicles reduce available cargo space, decreasing revenue per kilometre^63^. Cargo capacity is crucial when assessing the feasibility and suitability of these technologies in freight transport. As shown in Table 1, an average heavy BEV’s cargo capacity is 67% of a heavy conventional ICE truck, but advancements in battery technology are expected to increase it to 75% by 2050.

**Table 1:** Range of cargo capacity and refuelling time relative to conventional ICEs^22^.

Fuel—Powertrain	Weight class	Cargo capacity multiplier		Refuelling/recharging time multiplier	
		2020	2050	2020	2050
Natural gas-ICE	Small trucks	1.00	1.00	1.20	1.00
	Medium trucks	0.99	1.00	1.20	1.00
	Heavy trucks	0.95	1.00	1.20	1.00
Electricity—BEV	Small trucks	0.93	1.00	7.00	1.50
	Medium trucks	0.80	0.88	7.50	1.75
	Heavy trucks	0.67	0.75	8.00	2.00
Hydrogen—FCV	Small trucks	0.99	1.00	1.20	1.00
	Medium trucks	0.98	1.00	1.20	1.00
	Heavy trucks	0.96	1.00	1.20	1.00

The second one is increased recharging/refuelling time for zero-emission trucks. Unlike traditional Diesel ICEs, BEVs and FCVs take longer to recharge or refuel, leading to reduce fleet productivity^22^. This technological barrier^64^ has been recognised as a significant obstacle in adopting electric trucks^48,65^. Heavy BEVs, for example, take on average 8 times longer to recharge than conventional ICE vehicles take to refuel (see the full assumptions in Table 1). The expected availability factor for BEVs is lower than that of ICE vehicles, assuming one full charge every 24 h. However, advancements in battery technology are expected to improve BEVs’ efficiency and productivity, with estimated availability factors of around 37 thousand kilometres per year in 2020 and 47 thousand kilometres per year in 2050. These improvements will enable BEVs to travel further and recharge more quickly, contributing to increased efficiency and productivity for fleets utilising them.

Lastly, hesitancy in investing in new technologies is another intangible cost. The hesitancy in investing in new technologies can be closely tied to the concept of the hurdle rate, which is the minimum rate of return that an investment must generate to be considered viable^66^. The upfront costs of new technologies, such as BEVs and FCVs, are often high, which may discourage truck fleet operators from adopting them^48^. Previous studies on discount rates for the HGV sector suggest discount rate between 8.5 to 12%^66–69^. According to the Department of Transport^11^, the freight transport market is characterised by high levels of competition and relatively low profit margins for HGV operators. This, in combination with the volatile fuel prices, may suggest a higher required rate of return for investors, which could be reflected in a higher discount rate. Therefore, in this study, a discount rate of 12% is used for the HGV sector to account for these market conditions and associated risks.

### Scenario definition

The model runs through three main scenarios including a Reference and two mitigation scenarios:

- *Reference*: This scenario represents a business-as-usual situation where current trends in energy consumption and technology performance continue without any measures to address climate change. It serves as a benchmark for understanding future challenges and developments in long-term scenarios related to transitioning to a new energy system. It does not include specific targets for reducing CO_2_ emissions.
- *Net zero (NZ)*: This scenario introduces a carbon budget constraint. This means that the model produces energy system pathways for energy supply and demand in Ireland that align with a predetermined carbon budget target (see the details in reference^59^).
- *Net zero + intangible costs (NZI)*: In addition to the carbon budget constraint, NZI considers the activation of intangible costs. It explores the combined impact of all intangible costs.

Furthermore, three sensitivity cases examine the individual impacts of intangible costs. Each sensitivity case varies a single intangible cost by ± 30%. It is important to note that all other assumptions, including mobility demand levels, are assumed to follow the same projection across all scenarios.

## Data Availability

The TIMES-Ireland Model composed of excel files including the structure of the energy system, more than 300 commodities, more than 2000 specific technologies and their corresponding techno-economic parameters, and more than 150 constraints. It is publicly available on GitHub: https://github.com/MaREI-EPMG/times-ireland-model, last access: 12 Jan 2024.
